# Lack of Association between the Serotonin Transporter (*5-HTT*) and Serotonin Receptor (*5-HT2A*) Gene Polymorphisms with Smoking Behavior among Malaysian Malays

**DOI:** 10.3797/scipharm.1406-01

**Published:** 2014-07-18

**Authors:** Nur Iwani A Rozak, Imran Ahmad, Siew Hua Gan, Ruzilawati Abu Bakar

**Affiliations:** ^1^ Department of Pharmacology, School of Medical Sciences, Universiti Sains Malaysia, Kelantan, Malaysia.; ^2^ Department of Family Medicine, School of Medical Sciences, Universiti Sains Malaysia, Kelantan, Malaysia.; ^3^ Human Genome Centre, School of Medical Sciences, Universiti Sains Malaysia, Kelantan, Malaysia.

**Keywords:** 5-HTTLPR, 5HT_2A_, Genetic polymorphism, PCR-RFLP, Malaysian Malays male

## Abstract

An insertion/deletion polymorphism in the promoter region of the serotonin transporter gene (*5-HTTLPR*) and a polymorphism (rs6313) in the serotonin 2A receptor gene (*5-HT2A*) have previously been linked to smoking behavior. The objective of this study was to determine the possible association of the *5-HTTLPR* and *5-HT2A* gene polymorphisms with smoking behavior within a population of Malaysian male smokers (n=248) and non-smokers (n=248). The *5-HTTLPR* genotypes were determined using the polymerase chain reaction (PCR) and were classified as short (S) alleles or long (L) alleles. The *5HT2A* genotypes were determined using PCR-restriction fragment length polymorphisms (PCR-RFLP). No significant differences in the distribution frequencies of the alleles were found between the smokers and the non-smokers for the *5-HTTLPR* polymorphism (x^2^ = 0.72, P>0.05) or the *5HT2A* polymorphism (x^2^ = 0.73, P>0.05). This is the first study conducted on Malaysian Malay males regarding the association of *5-HTTLPR* and *5HT2A* polymorphisms and smoking behavior. However, the genes were not found to be associated with smoking behavior in our population.

## Introduction

Nicotine is the main addictive substance in cigarettes and is responsible for the development, as well as the maintenance, of the smoking addiction [1]. Cigarette smoking is a major preventable cause of disease. Genetic variables appear to play key roles in every aspect of nicotine addiction [2]. Therefore, genes involved in the pharmacodynamics and pharmacokinetics of nicotine are logical targets of nicotine addiction.

Serotonin (5-hydroxtryptamine, 5-HT) is a neurotransmitter derived from the amino acid tryptophan, and it has a wide range of central nervous system activities [3]. Polymorphisms affecting the serotonergic system, such as those in the serotonin transporter (5-HTT) and 5-HT genes, have been linked to smoking-related behaviors because nicotine increases serotonin release [4]. Therefore, variations in the serotonergic system may influence some aspects of smoking, such as mood variations during nicotine withdrawal [5]. One study showed that serotonin release was increased in the cortical region of the brain in rats treated with nicotine and that nicotine withdrawal seemed to be related to the subsequent decrease in serotonin [4].

5-HTT controls the duration and concentration of serotonin neurotransmission in the synaptic cleft [6]. 5-HTT is encoded by a single gene (SLC6A4) that is located on the long arm of chromosome 17 (17q12) [7]. The 5-HTT gene has been linked to psychological traits, such as anxiety-related personality traits [8] and depression [9] and these traits are also related to nicotine dependence [10, 11]. A 44 bp insertion or deletion polymorphism located in the serotonin transporter-linked polymorphic region (5-HTTLPR) was identified within this gene, resulting in two allelic variants, the long (L) and short (S) alleles, which alter the transcriptional efficiency of the 5-HTT gene [12]. The S allele has been associated with reduced serotonin uptake, leading to the hypothesis that individuals with the S allele are not prone to smoking [8]. This hypothesis is supported by two studies in Japanese and Chinese populations that found that individuals with the homozygous S genotype were less likely to initiate smoking behavior and could more easily stop smoking than others [13, 14]. However, the hypothesized role of the S allele in smoking behavior has not yet been confirmed; other studies that attempted to replicate these findings have obtained contradictory results [15–18], indicating that the influence of these genes may vary from one ethnicity to another.

The 5-HT receptor has been classified into seven groups (5-HT1-7); one of them, subgroup 5-HT2, can be further divided into three subclasses (2A, 2B, and 2C). The 5-HT2A gene is located on chromosome 13q14–q21 [19]. The 5HT2A gene has been associated with emotional disorders and alcohol dependence, both of which are related to smoking behavior [20]. One of the polymorphisms that have been described in this receptor is T102C, which involves the substitution of a cytosine residue with a thymine residue [19]. This polymorphism has been associated with the continuation of smoking behavior, but is less likely to be involved in the initiation of smoking behavior [21]. A study conducted in a Brazilian population found that individuals with the CC genotype are more likely to be current smokers than individuals with other genotypes. Interestingly, the opposite finding was obtained in Caucasian Australians; in this study, the occurrence of the TT genotype was associated with current smokers [22]. However, this finding has not been replicated in several other studies in different populations [23–25], indicating that the influence of these genes may vary from one ethnicity to another.

The present study investigated the possible associations between the 5-HTTLPR and 5-HT2A polymorphisms and the smoking behavior of Malaysian Malay males, which has not yet been investigated. To the best of our knowledge, this is the first study conducted on a Malaysian Malay male population regarding the association of 5-HTTLPR and 5-HT2A polymorphisms and smoking behavior. If such an association is established, it may explain why different individuals have different risks for nicotine dependence and why some individuals are able to quit smoking more easily, suggesting that specific interventional approaches should be used for certain individuals based on their genetic background.

## Results and Discussion

The average number of cigarettes per day of current smokers is less than 11. The genotype and allele frequencies of the 5-HTTLPR and 5-HT2A polymorphisms in smokers and non-smokers are given in Table 1. The frequencies of the 5-HTT alleles S/S, L/L, and heterozygous S/L in the non-smoker population were 39.1%, 11.3%, and 49.6%, respectively, whereas in the smokers, the frequencies of the S/S, L/L, and heterozygous S/L alleles were 41.1%, 12.9%, and 46.0%, respectively. The genotype frequencies for the 5-HT2A polymorphism in Malay smokers were 10.1% for CC, 46.8% for TT, and 43.1% for CT, whereas in the Malay non-smokers, the frequencies were 8.1% for CC, 46.4% for TT, and 45.6% for CT. No significant differences in the genotype frequencies of the 5-HTTLPR polymorphism (x^2^ = 0.73, P>0.05) and the 5-HT2A polymorphism (x^2^ = 0.72, P>0.05) were observed between the smoking group and the non-smoking group. The samples demonstrated Hardy-Weinberg equilibrium. Population stratification was not likely to be a confounder in this study because the samples were carefully limited to individuals with at least three generations of Malay descent.

Several studies previously examined the role of 5-HTT in smokers, but the results have been contradictory. We established that the 5-HTTLPR polymorphism was not associated with smoking behavior in the Malay population in Malaysia. This finding is consistent with five previous studies in Dutch [17], American [27], Austrian [15], Polish [16], and Greek [18] populations. Another study involving a Thai population also found no significant association between 5-HTTLPR polymorphisms and smoking behavior. However, individuals who carried the L allele were found to have higher-intensity smoking behaviors [25].

An association between 5-HTLLPR and smoking behavior was found in Japanese and Chinese populations. These studies suggested that the L allele was more likely to be carried by smokers than non-smokers, and smokers with homozygous S alleles were able to quit smoking more easily [13, 14]. Additionally, Lerman et al. [29] and Gerra et al. [31] reported that individuals with either homozygous or heterozygous S alleles were more likely to be dependent on nicotine than individuals with homozygous L alleles. The association of the 5-HTTLPR polymorphism with altered transcriptional activity might explain why different individuals exhibit different risks for nicotine dependence and why some individuals are able to quit more easily.

**Tab. 1.** Genotype and allele frequencies of the 5-HTTLPR and 5-HT2A with smoking behavior in the Malay Male population

Genotypes	Groups (n=496)		Statistics^a^	
	Smokers (n=248)	Non-smokers (n=248)	X^2^	P-value
5-HTTLPR				
SS (%)	102 (41.1)	97 (39.1)	0.734	0.693
LL (%)	32 (12.9)	28 (11.3)		
SL (%)	114 (46.0)	123 (49.6)		
Alleles				
S (%)	318 (64.1)	317 (63.9)		
L (%)	178 (35.9)	179 (36.0)		
5-HT2A				
CC (%)	25 (10.1)	20 (8.1)	0.724	0.696
TT (%)	116 (46.8)	115 (46.4)		
CT (%)	107 (43.1)	113 (45.6)		
Alleles				
C (%)	157 (31.7)	153 (30.8)		
T (%)	339 (68.3)	343 (69.2)		

^a^ Chi-square likelihood ratio test was used to compare the genotype frequencies.

Similar to the findings for 5-HTTLPR, our findings for the 5-HT2A polymorphism were consistent with several previous studies that found no association between the polymorphisms in the 5HT gene and smoking behavior [23–25]. A study on a Brazilian population found that the 5-HT2A polymorphism was related to smoking behavior; in particular, the CC genotype was more frequently found in current smokers than in former smokers or individuals who had never smoked [21]. However, this finding contradicts a study of Caucasian Australians that found an association between the TT genotype and current cigarette smoking status [22].

Tables 2 and 3 show the different studies that have investigated the association of the serotonin transporter and receptor with smoking behavior. Ethnic differences may contribute to the varied findings for the associations of the 5-HTTLPR and 5-HT2A polymorphisms with smoking behavior, as the distributions of certain alleles largely vary within different races. The L allele of 5-HTTLPR is more common among Polish populations [18], whereas the frequency of the L allele is lower in the Japanese population than in European, American, or African American populations [28]. By contrast, in Malay and Thai populations, the S allele is more common than the L allele. The T allele of the 5-HT2A gene is common in Brazilian and Asian populations, including the Malay, Thai, and Japanese populations. However, Caucasians and South Asians living in the United Kingdom have a higher C allele frequency [24]. This finding may be due to intermarriages between citizens of the United Kingdom that contribute to the inheritance of the C allele in South Asian descendants.

**Tab. 2.** Association studies of 5-HTTLPR in different ethnic groups with smoking behavior

Region	Sample size	Association with smoking behavior	Reference
Malaysia	248 smokers and 248 non-smokers	No	Current study
USA	268 smokers and 230 non-smokers	No	[27]
Japan	202 smokers, 82 non-smokers, and 103 ex-smokers	Long allele	[13]
USA	185 smokers	Short allele	[29]
Israel	190 smokers and 54 ex-smokers	Long allele	[30]
Italy	107 smokers and 103 non-smokers	Short allele	[31]
Austria	470 smokers and 419 non-smokers	No	[15]
Poland	149 smokers and 158 non-smokers	No	[16]
Denmark	327 smokers, 642 non-smokers, and 396 ex-smokers	No	[17]
China	144 smokers and 135 non-smokers	Long allele	[14]
Sweden	80 smokers and 120 non-smokers	Long allele	[32]
Greece	172 smokers and 254 non-smokers	No	[18]
Thailand	200 smokers and 111 non-smokers	No	[25]
USA	177 smokers, 458 non-smokers, and 124 ex-smokers	Short allele	[33]

**Tab. 3.** Association studies of 5-HT2A in different ethnic groups with smoking behavior

Region	Sample size	Association with smoking behavior	Reference
Malaysia	248 smokers and 248 non-smokers	No	Current study
Japan	82 smokers and 125 non-smokers	No	[23]
Caucasian Australia	47 smokers and 85 non-smokers	Yes	[22]
Thailand	200 smokers and 111 non-smokers	No	[25]
Brazil	193 smokers and 432 non-smokers	Yes	[21]

Additionally, the discrepancies observed in previous studies on the results of the 5-HTTLPR and 5-HT2A polymorphisms may be attributed to gene-gene interactions. These two genes may be linked to other genes that determine smoking behavior. However, the degree of linkage between 5-HTTLPR or 5-HT2A and the putative genes may again vary from ethnicity to ethnicity. This difference may explain why polymorphisms in 5-HTTLPR and 5-HT2A have no association with smoking behavior in the Malaysian population but may be associated in other populations.

Variations in subject recruitment methods may also explain discrepant results between studies. Intermarried ancestors may become confounding factors in these studies; therefore, subjects should be restricted to a single race. Additionally, sample sizes also vary between studies. The study design should clearly define which individuals are smokers. Some studies may include ex-smokers as smokers [21], whereas other studies may define ex-smokers as non-smokers [16], leading to further variations. However, the correlation between genetics and smoking behavior and the smoking cessation success rate may affect the overall outcome of the correlation.

Our study is subjected to some limitations. First, our study did not focus on the age groups where all subjects between 18 and 50 years old were included which may lead to some variability. In addition, the average age for the non-smokers was in the 20s while that for the smokers was in the 30s. A possible reason for this discrepancy could be attributed to the fact that most non-smokers were recruited from institutes of higher education (Kota Bharu Institute of Teacher Education and Universiti Sains Malaysia). On the other hand, the majority of smokers were recruited from various locations in Kota Bharu. This supports the findings that community and school level variables are predictive of adolescent smoking [34]. Secondly, we did not consider the effects of negative environment and social conditions of the subjects such as stressful life events and schooling level which may also interfere with the prevalence of the 5-HT polymorphism.

The study of the association between genetics and smoking behavior is complex and involves many known and unknown genes, as well as clinical and environmental factors, which may interact with each other. Any finding of an association between gene polymorphisms and smoking behavior may only be true for the population studied. Future research should investigate other possible gene polymorphisms using a larger sample size across cultural and ethnic groups to identify the true universal contributors that influence smoking behavior.

## Experimental

### Subjects

A total of 496 Malaysian Malay male subjects, both smokers (n=248) and non-smokers (n=248), between 18 and 50 years old were selected. Smoking was defined as having smoked more than 100 cigarettes in a lifetime and being a current smoker at the time of the study. The subjects were confirmed to be of the Malay ethnic group for at least three generations. Subjects having a serious disorder or a previous history of a mental disorder were excluded from this study. Only males were recruited to avoid inter-individual variations that may arise due to sex differences and hormonal changes commonly shown by females. The demographic data of the subjects are shown in Table 4. All participants gave written informed consent before they were enrolled, and the study was approved by the Ethics Committee (ethical no: USMKK/PPP/JEPeM[233.3.(05)]/Amend.01) of the School of Medical Sciences, Universiti Sains Malaysia, which complies with the Declaration of Helsinki.

### DNA Extraction

Three milliliters of venous blood were drawn into a sterile tube containing EDTA and stored at -20°C until the isolation of genomic DNA. Genomic DNA was isolated from the blood using the QIAamp DNA Blood Mini Kit (Qiagen, USA). DNA purity and concentration were determined by measuring the absorbance at 280 nm using a Biophotometer Uvette spectrophotometer (Eppendorf, Germany).

**Tab. 4.** Demographic data subjects

	Smokers (n=248)	Non-smokers (n=248)	*p* value
	Mean (SD)	Mean (SD)	
Age (years)^a^	33.50 (19.0)^c^	24.00 (16.0)^c^	0.000^a^
Height (m)^b^	168.5 (0.06)	168.64 (0.06)	0.307^b^
Weight (kg)^b^	69.24 (12.45)	69.58 (12.32)	0.760^b^
Body mass index (kg/m^2^)^b^	24.50 (4.05)	24.47 (4.16)	0.945^b^
Brachial systolic blood pressure (mm/Hg)^b^	126.89 (11.81)	130.27 (13.69)	0.001^b^
Brachial diastolic blood pressure (mm/Hg)^b^	75.42 (9.91)	75.91 (9.45)	0.578^b^

^a^ Mann Whitney test;

^b^ Independent-samples T-test;

^c^ Median (IQR).

### Molecular Analysis

**Fig. 1.**
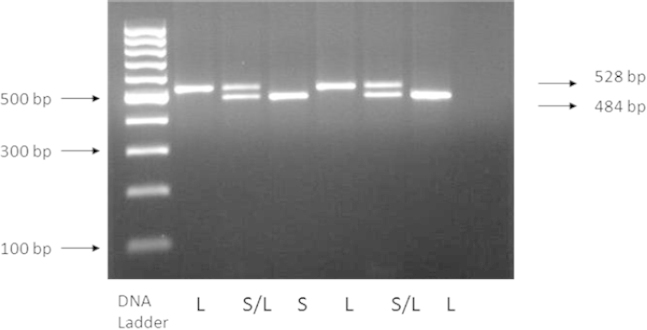
PCR products for amplification using 5-HTTLPR primers. Lane 1 and 4 show homozygous long allele (L), lane 2 and 5 show heterozygous short-long allele (S/L), while lane 3 and 6 show homozygous short allele (S)

Genotyping of the 5-HTTLPR gene polymorphism was performed by using the polymerase chain reaction (PCR) as previously described by Heils et al. [12] with some slight modifications. The PCR reaction was performed in a final volume of 25 μ;l containing 60 ng of genomic DNA, 200 μ;M dNTP mix, 0.24 μ;M of each primer (forward 5'-GGCGTTGCCGCTCTGAATGC-3’ and reverse 5’-GAGGGACTGAGCTGGACAACCAC-3'), 0.75 mM magnesium chloride (MgCl2), 1x ammonium sulfate buffer [(NH4)¬2SO4] (Fermentas, Vilnius, Lithuania), and 1.25 U Taq polymerase (Fermentas, Vilnius, Lithuania). After an initial incubation at 95°C for 2 min, the PCR products were amplified for 35 cycles of denaturation at 95°C for 30 sec, annealing at 62°C for 30 sec, and extension at 72°C for 1 min. The final extension step lasted 7 min at 72°C. The PCR products were then resolved using a 2% agarose gel and were visualized under UV light. Each gel contained one lane of a 100 bp ladder to identify the 528 bp fragment, designated as the L allele, and the 484 bp fragment, designated as the S allele (Fig. 1).

**Fig. 2.**
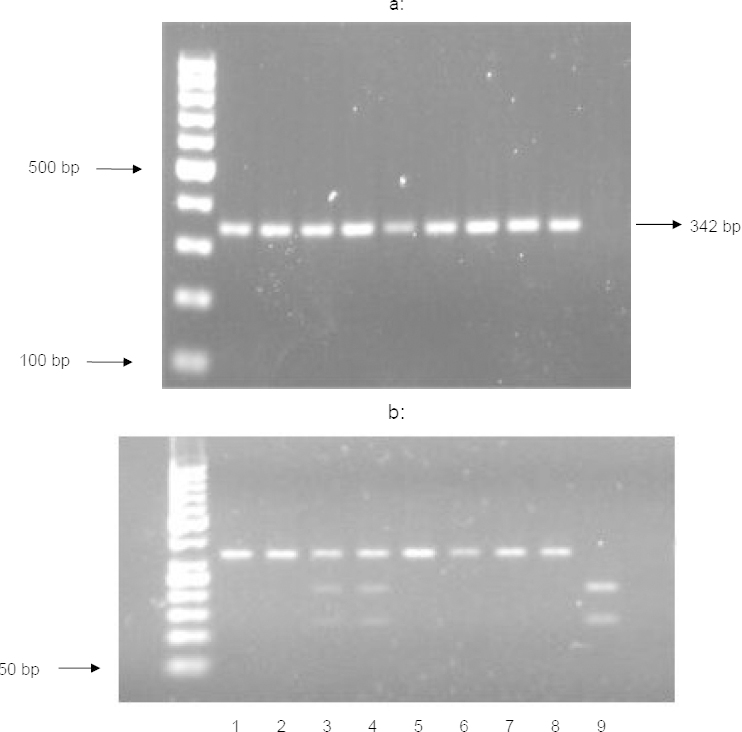
a: PCR products for amplification using 5-HT2A primers, b: PCR-RFLP result after digestion with *Hpa* II restriction enzyme. Lane 1, 2, 5, 6, 7, 8 show mutant allele with 342 bp. Lane 9 shows wild type allele with 126 and 216 bp fragments and Lane 3 and 4 show wild type-mutant alleles

Genotyping of the 5-HT2A polymorphism (*rs6313*) was performed as previously described [26], with several modifications. PCR was performed in a 25 μl reaction containing 78 ng of genomic DNA, 100 μM of each dNTP, 1.5 mM MgCl2, 0.25 uM of each primer (forward 5'-TGTGCTACAAGTTCTGGCTT-3' and reverse 5'-GTGCAGTTTTTCTCTAGGG-3'), and 0.75 U DNA Taq polymerase (Fermentas, Vilnius, Lithuania). After an initial denaturation at 95°C for 3 min, the PCR products were amplified for 35 cycles of 1 min at 94°C, 45 s at 53°C, and 1 min at 72°C, followed by a final extension step of 10 min at 72°C. The 342 bp PCR product was digested with Hpa II (New England Biolabs, MA, USA) for 3 h at 37°C. The digestion products were then resolved on a 2% agarose gel containing ethidium bromide and were visualized under UV light. The 102T mutant remained uncut (342 bp), whereas the wild type 102C allele was digested into two bands of 216 and 126 bp; three fragments of 342, 216, and 126 bp were observed for samples containing both the wild type and mutant alleles (Fig. 2).

### Statistical Analysis

The allele and genotype frequencies were calculated to equilibrium using the Hardy– Weinberg equation (p^2^ - 2pq - q^2^ = 1). The significance of the allele frequency or the genotype distribution among the volunteers with different smoking habits was examined using a non-parametric chi-square test. A P<0.05 was considered statistically significant. All statistical analyses were performed using the SPSS package version 20.0 (IBM, Armonk, NY).

## Conclusion

The 5-HTTLPR and 5-HT2A gene polymorphisms were not found to be associated with smoking behavior in Malaysian Malay males. The study of the association between genetics and smoking behavior is complex and is dependent on many factors, making the association unique to each population.

## References

[1] Henningfield JE Miyasato K Jasinski DR Abuse liability and pharmacodynamic characteristics of intravenous and inhaled nicotine. J Pharmacol Exp Ther. 1985; 234: 1–12. http://www.ncbi.nlm.nih.gov/pubmed/4009494 4009494

[2] Li MD Cheng R Ma JZ Swan GE A meta-analysis of estimated genetic and environmental effects on smoking behavior in male and female adult twins. Addiction. 2003; 98: 23–31. http://dx.doi.org/10.1046/j.1360-0443.2003.00295.x 12492752 10.1046/j.1360-0443.2003.00295.x

[3] Kuo CL Wang RB Shen LJ Lien LL Lien EJ G-protein coupled receptors: SAR analyses of neurotransmitters and antagonists. J Clin Pharm Ther. 2004; 29: 279–298. http://dx.doi.org/10.1111/j.1365-2710.2004.00563.x 15153091 10.1111/j.1365-2710.2004.00563.x

[4] Ribeiro EB Bettiker RL Bogdanov M Wurtman RJ Effects of systemic nicotine on serotonin release in rat brain. Brain Res. 1993; 621: 311–318. http://dx.doi.org/10.1016/0006-8993(93)90121-3 8242344 10.1016/0006-8993(93)90121-3

[5] Tyndale RF Genetics of alcohol and tobacco use in humans. Ann Med. 2003; 35: 94–121. http://dx.doi.org/10.1080/07853890310010014 12795339 10.1080/07853890310010014

[6] Uhl GR Johnson PS Neurotransmitter transporters: three important gene families for neuronal function. J Exp Biol. 1994; 196: 229–236. http://www.ncbi.nlm.nih.gov/pubmed/7823024 7823024 10.1242/jeb.196.1.229

[7] Ramamoorthy S Bauman AL Moore KR Han H Yang-Feng T Chang AS Antidepressant- and cocaine-sensitive human serotonin transporter: molecular cloning, expression, and chromosomal localization. Proc Natl Acad Sci U S A. 1993; 90: 2542–2546. http://dx.doi.org/10.1073/pnas.90.6.2542 7681602 10.1073/pnas.90.6.2542 PMC46124

[8] Lesch KP Bengel D Heils A Sabol SZ Greenberg BD Petri S Association of anxiety-related traits with a polymorphism in the serotonin transporter gene regulatory region. Science. 1996; 274: 1527–15231. http://dx.doi.org/10.1126/science.274.5292.1527 8929413 10.1126/science.274.5292.1527

[9] Collier DA Stober G Li T Heils A Catalano M Di Bella D A novel functional polymorphism within the promoter of the serotonin transporter gene: possible role in susceptibility to affective disorders. Mol Psychiatry. 1996; 1: 453–460. http://www.ncbi.nlm.nih.gov/pubmed/9154246 9154246

[10] Audrain J Lerman C Gomez-Caminero A Boyd NR Orleans CT The Role of Trait Anxiety in Nicotine Dependence. J App Biobehav Res. 1998; 3: 29–42. http://dx.doi.org/10.1111/j.1751-9861.1998.tb00042.x

[11] Lerman C Audrain J Orleans CT Boyd R Gold K Main D Investigation of mechanisms linking depressed mood to nicotine dependence. Addict Behav. 1996; 21: 9–19. http://dx.doi.org/10.1016/0306-4603(95)00032-1 8729703 10.1016/0306-4603(95)00032-1

[12] Heils A Teufel A Petri S Stober G Riederer P Bengel D Allelic variation of human serotonin transporter gene expression. J Neurochem. 1996; 66: 2621–2624. http://dx.doi.org/10.1046/j.1471-4159.1996.66062621.x 8632190 10.1046/j.1471-4159.1996.66062621.x

[13] Ishikawa H Ohtsuki T Ishiguro H Yamakawa-Kobayashi K Endo K Lin YL Association between serotonin transporter gene polymorphism and smoking among Japanese males. Cancer Epidemiol Biomarkers Prev. 1999; 8: 831–833. http://www.ncbi.nlm.nih.gov/pubmed/10498403 10498403

[14] Chu SL Xiao D Wang C Jing H Association between 5-hydroxytryptamine transporter gene-linked polymorphic region and smoking behavior in Chinese males. Chin Med J (Engl). 2009; 122: 1365–1368. http://www.ncbi.nlm.nih.gov/pubmed/19567154 19567154

[15] Trummer O Koppel H Wascher TC Grunbacher G Gutjahr M Stanger O The serotonin transporter gene polymorphism is not associated with smoking behavior. Pharmacogenomics J. 2006; 6: 397–400. http://dx.doi.org/10.1038/sj.tpj.6500389 16702982 10.1038/sj.tpj.6500389

[16] Sieminska A Buczkowski K Jassem E Tkacz E Lack of association between serotonin transporter gene polymorphism 5-HTTLPR and smoking among Polish population: a case-control study. BMC Medical Genetics. 2008; 9: 76 http://dx.doi.org/10.1186/1471-2350-9-76 18691405 10.1186/1471-2350-9-76 PMC2529278

[17] Rasmussen H Bagger Y Tanko LB Christiansen C Werge T Lack of association of the serotonin transporter gene promoter region polymorphism, 5-HTTLPR, including rs25531 with cigarette smoking and alcohol consumption. Am J Med Genet B Neuropsychiatr Genet. 2009; 150B: 575–580. http://dx.doi.org/10.1002/ajmg.b.30880 18951433 10.1002/ajmg.b.30880

[18] Iordanidou M Tavridou A Petridis I Kyroglou S Kaklamanis L Christakidis D Association of polymorphisms of the serotonergic system with smoking initiation in Caucasians. Drug Alcohol Depend. 2009; 108: 70–76. http://dx.doi.org/10.1016/j.drugalcdep.2009.11.015 20060656 10.1016/j.drugalcdep.2009.11.015

[19] Peroutka SJ Serotonin Receptor Variants in Disease: New Therapeutic Opportunities? Ann N Y Acad Sci. 1998; 861: 16–25. http://dx.doi.org/10.1111/j.1749-6632.1998.tb10168.x 10.1111/j.1749-6632.1998.tb10168.x 9928234

[20] Polina ER Contini V Hutz MH Bau CH The serotonin 2A receptor gene in alcohol dependence and tobacco smoking. Drug Alcohol Depend. 2009; 101: 128–131. http://dx.doi.org/10.1016/j.drugalcdep.2008.11.001 19111403 10.1016/j.drugalcdep.2008.11.001

[21] do Prado-Lima PA Chatkin JM Taufer M Oliveira G Silveira E Neto CA Polymorphism of 5HT2A serotonin receptor gene is implicated in smoking addiction. Am J Med Genet B Neuropsychiatr Genet. 2004; 128B: 90–93. http://dx.doi.org/10.1002/ajmg.b.30004 15211639 10.1002/ajmg.b.30004

[22] White MJ Young RM Morris CP Lawford BR Cigarette smoking in young adults: the influence of the HTR2A T102C polymorphism and punishment sensitivity. Drug Alcohol Depend. 2010; 114: 140–146. http://dx.doi.org/10.1016/j.drugalcdep.2010.08.014 21035274 10.1016/j.drugalcdep.2010.08.014

[23] Terayama H Itoh M Fukunishi I Iwahashi K The serotonin-2A receptor polymorphism and smoking behavior in Japan. Psychiatr Genet. 2004; 14: 195–197. http://dx.doi.org/10.1097/00041444-200412000-00005 15564892 10.1097/00041444-200412000-00005

[24] Huang S Cook DG Hinks LJ Chen XH Ye S Gilg JA CYP2A6, MAOA, DBH, DRD4, and 5HT2A genotypes, smoking behaviour and cotinine levels in 1518 UK adolescents. Pharmacogenet Genomics. 2005; 15: 839–850. http://dx.doi.org/10.1097/01213011-200512000-00002 16272956 10.1097/01213011-200512000-00002

[25] Suriyaprom K Phonrat B Chuensumran U Tungtrongchitr A Tungtrongchitr R Association of HTTLPR and 5-HT(2)A T102C polymorphisms with smoking characteristics and anthropometric profiles of Thai males. Genet Mol Res. 2012; 11: 4360–4369. http://dx.doi.org/10.4238/2012.October.15.2 23096916 10.4238/2012.October.15.2

[26] Warren JT Jr Peacock ML Rodriguez LC Fink JK An MspI polymorphism in the hyman serotonin receptor gene (HTR2): detection by DGGE and RFLP analysis. Hum Mol Genet. 1993; 2: 338 http://dx.doi.org/10.1093/hmg/2.3.338 7684645 10.1093/hmg/2.3.338

[27] Lerman C Shields PG Audrain J Main D Cobb B Boyd NR The role of the serotonin transporter gene in cigarette smoking. Cancer Epidemiol Biomarkers Prev. 1998; 7: 253–255. http://www.ncbi.nlm.nih.gov/pubmed/9521442 9521442

[28] Gelernter J Kranzler H Cubells JF Serotonin transporter protein (SLC6A4) allele and haplotype frequencies and linkage disequilibria in African- and European-American and Japanese populations and in alcohol-dependent subjects. Hum Genet. 1997; 101: 243–246. http://dx.doi.org/10.1007/s004390050624 9402979 10.1007/s004390050624

[29] Lerman C Caporaso NE Audrain J Main D Boyd NR Shields PG Interacting effects of the serotonin transporter gene and neuroticism in smoking practices and nicotine dependence. Mol Psychiatry. 2000; 5: 189–192. http://dx.doi.org/10.1038/sj.mp.4000672 10822347 10.1038/sj.mp.4000672

[30] Kremer I Bachner-Melman R Reshef A Broude L Nemanov L Gritsenko I Association of the serotonin transporter gene with smoking behavior. Am J Psychiatry. 2005; 162: 924–930. http://dx.doi.org/10.1176/appi.ajp.162.5.924 15863794 10.1176/appi.ajp.162.5.924

[31] Gerra G Garofano L Zaimovic A Moi G Branchi B Bussandri M Association of the serotonin transporter promoter polymorphism with smoking behavior among adolescents. Am J Med Genet B Neuropsychiatr Genet. 2005; 135B: 73–78. http://dx.doi.org/10.1002/ajmg.b.30173 15806583 10.1002/ajmg.b.30173

[32] Nilsson KW Oreland L Kronstrand R Leppert J Smoking as a product of gene-environment interaction. Ups J Med Sci. 2009; 114: 100–107. http://dx.doi.org/10.1080/03009730902833406 19396697 10.1080/03009730902833406 PMC2852756

[33] Hu S Brody CL Fisher C Gunzerath L Nelson ML Sabol SZ Interaction between the serotonin transporter gene and neuroticism in cigarette smoking behavior. Mol Psychiatry. 2000; 5: 181–188. http://dx.doi.org/10.1038/sj.mp.4000690 10822346 10.1038/sj.mp.4000690

[34] Kelly AB O'Flaherty M Connor JP Homel R Toumbourou JW Patton GC Williams J The influence of parents, siblings and peers on pre- and early-teen smoking: A multilevel model. Drug Alcohol Rev. 2011; 30: 381–387. http://dx.doi.org/10.1111/j.1465-3362.2010.00231.x 21355905 10.1111/j.1465-3362.2010.00231.x

